# A survey of tobacco dependence treatment services in 121 countries

**DOI:** 10.1111/add.12172

**Published:** 2013-04-10

**Authors:** Hembadoon Piné-Abata, Ann McNeill, Rachael Murray, Asaf Bitton, Nancy Rigotti, Martin Raw

**Affiliations:** 1 UK Centre for Tobacco Control Studies, Division of Epidemiology and Public Health, University of NottinghamNottingham, UK; 2 UK Centre for Tobacco Control Studies, National Addiction Centre, Institute of Psychiatry, King's College LondonLondon, UK; 3 Division of General Medicine, Brigham and Women's Hospital, Department of Health Care Policy, Harvard Medical SchoolBoston, MA, USA; 4 Department of Medicine, Harvard Medical SchoolBoston, MA, USA; 5 Tobacco Research and Treatment Center, General Medicine Division, Massachusetts General HospitalBoston, MA, USA

**Keywords:** FCTC, Article 14, Article 14 guidelines, survey, tobacco dependence treatment, treatment systems

## Abstract

**Aims:**

To report progress among Parties to the World Health Organization (WHO) Framework Convention on Tobacco Control (FCTC) in developing tobacco dependence treatment systems in accordance with FCTC Article 14 and the Article 14 guidelines recommendations.

**Design:**

Cross-sectional study.

**Setting:**

Electronic survey from December 2011 to August 2012.

**Participants:**

One hundred and sixty-three of the 174 Parties to the FCTC at the time of our survey.

**Measurements:**

The 51-item questionnaire contained 21 items specifically on treatment systems. Questions covered the availability of basic treatment infrastructure and national cessation support systems.

**Findings:**

We received responses from 121 (73%) of the 166 countries surveyed. Fewer than half of the countries had national treatment guidelines (*n* = 53, 44%), a government official responsible for tobacco dependence treatment (*n* = 49, 41%), an official national treatment strategy (*n* = 53, 44%) or provided tobacco cessation support for health workers (*n* = 55, 46%). More than half encouraged brief advice in existing health care services (*n* = 68, 56%), while only 44 (36%) had quitlines and only 20 (17%) had a network of treatment support covering the whole country. Low- and middle-income countries had less tobacco dependence treatment provision than high-income countries.

**Conclusion:**

Most countries, especially low- and middle-income countries, have not yet implemented the recommendations of FCTC Article 14 or the FCTC Article 14 guidelines.

## Introduction

Article 14 of the 2005 World Health Organization (WHO) Framework Convention on Tobacco Control (FCTC) 1 requires Parties to take effective measures to promote cessation of tobacco use and adequate treatment for tobacco dependence. In November 2010 the fourth Conference of the Parties to the FCTC adopted guidelines for the implementation of Article 14 2. These guidelines amount to official policy on tobacco dependence treatment for Parties to the Convention and, *inter alia*, outline the basic infrastructure needed to support tobacco cessation and key components of a national system to help tobacco users quit. Tobacco dependence treatment is defined by the Article 14 guidelines as: ‘The provision of behavioural support or medications, or both, to tobacco users, to help them stop their tobacco use’ 2.

The basic infrastructure elements to support tobacco cessation include the following 2:

- A focal point or national coordinating mechanism
- A national cessation strategy
- National treatment guidelines
- Programmes to encourage health care workers who use tobacco to stop and support to help them do so
- National training standards
- The use, as far as possible, of existing infrastructure including but not limited to HIV/AIDS, tuberculosis and related services
- Mandatory reporting of tobacco use in all medical notes
- Establishing a sustainable source of funding for cessation support

The key components of a national cessation support system include 2:

- Mass communication and education programmes to encourage cessation
- Brief advice integrated into all health care systems
- Quitlines
- Access to affordable medications
- Specialized tobacco dependence treatment services

The Article 14 guidelines suggest that Parties develop cessation support in a stepwise order: first establish basic infrastructure elements; then address the issue in health-care workers; integrate brief advice into existing health-care systems; and finally develop treatment support, including quitlines and specialist services. Finally, the guidelines state that treatment should be widely available, accessible and affordable and that cessation and treatment strategies should be monitored.

In this paper we report the results of a survey whose objective was to review the state of tobacco dependence treatment systems and national treatment guidelines in Parties to the FCTC, in order to gauge progress in implementing Article 14 and its guidelines. The survey builds on a previous survey, the results of which were published in 2009 3,4. We report here the treatment system results; the results on national treatment guidelines are reported in a separate paper 5.

## Methods

There were 174 Parties to the FCTC, including the European Union (EU), in addition to its member countries, at the time our survey began in December 2011. We excluded the EU, and we were unable to find contacts in 10 Parties (two high-income, three upper-middle-income, two lower-middle-income and three low-income countries), thus 163 Parties were surveyed. The United Kingdom, which is a Party, consists of four countries, England, Northern Ireland, Scotland and Wales, each with separate health care systems and treatment guidelines, so we surveyed all four individually. Our final sample therefore consisted of 163 FCTC Parties or 166 countries. Our sample of contacts—a mixture of treatment specialists, FCA members and government officials—was identified from those used in the previous survey 3,4, our own contacts and recommendations from a range of organizations. We endeavoured to identify people who were likely to be knowledgeable about tobacco cessation and treatment provision in their countries.

We e-mailed 166 people starting in December 2011, inviting them to participate in our survey by either clicking on a link to the online survey or by completing an attached Word questionnaire (offered in English, French and Spanish). We followed-up non-responders with reminder e-mails in January, February, April and May 2012.

The questionnaire contained 51 items, 21 specifically on treatment, and is available in the online version of this paper. Responses to the question on availability and licensing of medications proved difficult to interpret, partly because of its construction. Thus, we also e-mailed manufacturers of the four principal cessation medications and asked them to provide data on availability of their medications internationally. These data were used as the base to check respondents' awareness of availability and perceived affordability.

Countries were categorized by WHO region 6 and World Bank income level in August 2012, based on Gross National Income (GNI) per capita in 2011. The categories are low-income ($1025 or less), lower-middle-income ($1026–4035), upper-middle-income ($4036–12 475) and high-income ($12 476 or more) 7. We reported the distribution of our respondents by WHO region and World Bank income category and present the main findings broken down by income category.

## Results

**Figure 1:**
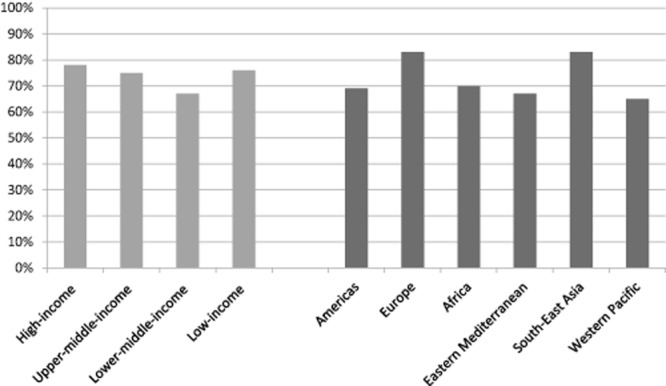
Survey response rates by region and World Bank income level of respondent countries

We received responses from 121 of the 166 countries surveyed, a response rate of 73%. Our United Arab Emirates contact completed the survey only for Abu Dhabi. The highest and lowest response rates by region were 83% in Europe and South-East Asia, and 65% in the Western Pacific region (Fig. 1). By World Bank income level, the highest response rate was 78% in high-income countries, and the lowest was 67% in lower-middle-income countries.

### Basic infrastructure elements

According to respondents, fewer than half of countries had an official national treatment strategy (44%), a government official responsible for tobacco dependence treatment (41%) or national tobacco treatment guidelines (44%). Only one-fifth of countries had a clearly identified treatment budget, and 22% did not monitor the use of treatment services (Table 1); just under half offered tobacco cessation assistance to health workers (46%). Twenty-two per cent of countries mandated the recording of patients' tobacco use in medical notes. Just over a quarter of countries had national training standards.

**Table 1:** Basic infrastructure elements by World Bank income level of respondent countries

	% Yes (n)				
	
Question	All (n *=* 121)	High (n *=* 36)	UM (n *=* 36)	LM (n *=* 30)	Low (n *=* 19)
Does your country have an official national treatment strategy?	44 (53)	58 (21)	47 (17)	33 (10)	26 (5)
Is there an officially identified person who is responsible for tobacco dependence treatment?	41 (49)	47 (17)	44 (16)	40 (12)	21 (4)
Does your country have national guidelines for the treatment of tobacco dependence?	44 (53)	75 (27)	42 (15)	30 (9)	11 (2)
Have a clearly identified budget for treatment?	20 (24)	36 (13)	17 (6)	17 (5)	0 (0)
Does your country monitor the use of treatment services?	22 (27)	42 (15)	25 (9)	10 (3)	0 (0)
Does your country offer help to health care workers to stop using tobacco?	46 (55)	56 (20)	47 (17)	40 (12)	32 (6)
Does your country have mandatory recording of patients' tobacco use status in medical notes?	22 (26)	28 (10)	25 (9)	13 (4)	16 (3)
Does your country have national training standards?	26 (31)	42 (15)	25 (9)	13 (4)	16 (3)

LM = lower-middle-income; UM = upper-middle-income. Missing data ranged from zero to 1%.

### National cessation support system

Just over half of countries ran anti-tobacco mass media campaigns (54%) or encouraged the provision of brief advice in existing services (56%) (Table 2).

**Table 2:** Components of the national cessation support system by World Bank income level of respondent countries

	% Yes (n)				
	
Question	All (n *=* 121)	High (n *=* 36)	UM (n *=* 36)	LM (n *=* 30)	Low (n *=* 19)
Does your country run mass media campaigns promoting cessation?	54 (65)	69 (25)	50 (18)	43 (13)	47 (9)
Does your country promote/encourage brief advice in existing services?	56 (68)	56 (20)	50 (18)	60 (18)	63 (12)
Does your country have a telephone quitline?	36 (44)	75 (27)	28 (10)	20 (6)	5 (1)
• Is it free to callers calling in?	73 (32)	74 (20)	60 (6)	83 (5)	100 (1)
• Does it have people answering always or almost always?	80 (35)	85 (23)	60 (6)	83 (5)	100 (1)
• Does it offer multiple sessions with counsellors calling back offering ongoing support?	56 (24)	70 (19)	30 (3)	33 (2)	0 (0)
• Does it refer to local specialist treatment services?	86 (38)	85 (23)	100 (10)	67 (4)	100 (1)
• Does it offer information about tobacco cessation medications?	80 (35)	85 (23)	70 (7)	67 (4)	100 (1)
• Does it offer tobacco cessation medications to callers?	21 (9)	22 (6)	10 (1)	33 (2)	0 (0)
Countries with nation wide specialized tobacco dependence treatment facilities	17 (20)	36 (13)	19 (7)	0 (0)	0 (0)
Countries with treatment facilities but only in selected areas	51 (62)	50 (18)	56 (20)	57 (17)	37 (7)
Countries with no specialized treatment at all	32 (39)	14 (5)	25 (9)	43 (13)	63 (12)

LM = lower-middle-income; UM = upper-middle-income. Missing data ranged from zero to 1%. The base for the quitline questions (in bullet points) for all countries was the 44 countries with quitlines and for the respective income categories was the number of countries having quitlines in each category.

#### Quitlines

Respondents in 36% of countries (44 of 121) reported having quitlines. The majority of these were free to callers and had people (rather than machines) answering calls most of the time. More than half offered multiple sessions with call-back offering support, while 86% referred callers to local specialist treatment services and 80% offered information on tobacco cessation medications. Tobacco cessation medications were offered to callers by one-fifth of quitlines.

#### Specialist treatment facilities

Respondents in one-third of countries reported having no specialized treatment facilities at all; just over half had treatment support in selected areas, while 17% had a network of treatment support which covered the whole country (Table 2).

#### Access to help

**Figure 2:**
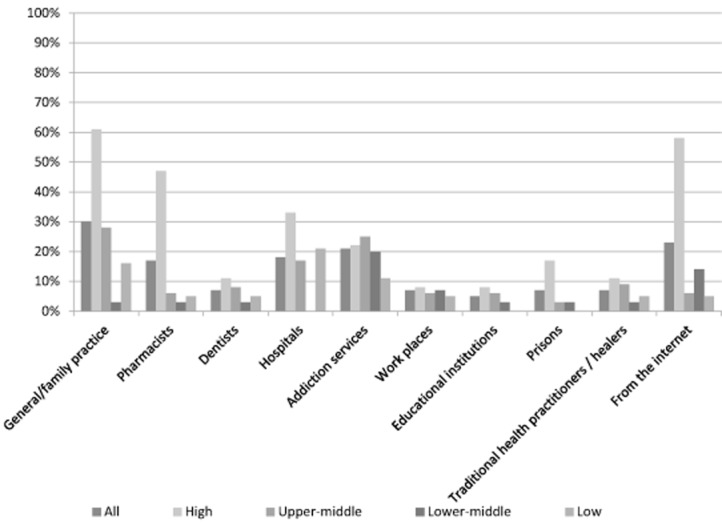
Can tobacco users obtain help easily to stop in the following settings? (Overall responses and responses by World Bank income level of respondent countries). The bars show the proportion answering ‘Yes, easily’ (Yes); the other responses were ‘Not easily’ and ‘No’

Respondents in almost one-third (30%) of countries indicated that tobacco users could obtain help easily in a general/family practice setting, while 17% said the same for pharmacists, 7% for dentists, 18% for hospitals and 23% from the internet (Fig. 2).

#### Access to medications

Based on manufacturer data, varenicline and nicotine replacement therapy (NRT) were the most widely available medications, being available in just over half of the respondent countries (Table 3). Respondents' awareness that medications were available ranged from 67% with cytisine to 100% with bupropion.

**Table 3:** Availability of medications by World Bank income level of respondent countries

	Availability according to manufacturer (n)					Respondents' awareness of medication availability % (n/base)
		
Medication	All (n *=* 121)	High (n *=* 36)	UM (n *=* 36)	LM (n *=* 30)	Low (n *=* 19)	All
NRT	51 (62)	97 (35)	61 (22)	17 (5)	0 (0)	92 (57/62)
Bupropion	17 (20)	42 (15)	14 (5)	0 (0)	0 (0)	100 (20/20)
Varenicline	52 (63)	83 (30)	58 (21)	33 (10)	11 (2)	86 (54/63)
Cytisine	10 (12)	3 (1)	17 (6)	13 (4)	5 (1)	67 (8/12)

LM = lower-middle-income; NRT = nicotine replacement therapy; UM = upper-middle-income. The bases used in the final column were the ‘*n*’ values given for the respective medications in the second column.

We asked respondents if a medication was ‘easily affordable to most tobacco users’. In countries whose respondents knew that the medication was available, cytisine was reported to be easily affordable in all countries, while varenicline was said to be the least easily affordable (Table 4).

**Table 4:** Affordability of medications by World Bank income level of respondent countries

	Affordability of medications known to be available % (n/base)				
	
Medication	All	High	UM	LM	Low
NRT	81 (46/57)	94 (32/34)	60 (12/20)	67 (2/3)	–
Bupropion	70 (14/20)	80 (12/15)	40 (2/5)	–	–
Varenicline	48 (26/54)	64 (18/28)	32 (6/19)	29 (2/7)	0 (0)
Cytisine	100 (8/8)	100 (1/1)	100 (4/4)	100 (2/2)	100 (1/1)

LM = lower-middle-income; NRT = nicotine replacement therapy; UM = upper-middle-income. The bases used in the second column were the ‘*n*’ values for respondents' awareness of availability of the respective medications given in Table 3.

#### Relationship between treatment provision and Parties' World Bank income level

In almost all cases, low- and middle-income countries had fewer basic infrastructure elements than high-income countries (Table 1). Fifty-eight per cent of high-income countries had an official national treatment strategy, while just over a quarter of low-income countries did. Just under half of high-income countries had an official responsible for treatment compared to only a fifth of low-income countries. Three-quarters of high-income countries had treatment guidelines as opposed to just 11% of low-income countries, and just over a third of high-income countries had a clearly identified treatment budget compared with no low-income countries.

A similar picture was observed with most components of national cessation support systems. More than two-thirds of high-income countries ran mass media campaigns promoting cessation, compared with fewer than half of low-income countries, and 75% of high-income countries provided quitlines compared with 5% of low-income countries. Finally, 36% of high-income countries provided specialist treatment facilities covering the whole country, whereas no low- or lower-middle-income country did, and only 14% of high-income countries provided no specialist treatment facilities at all, compared with 63% of low-income countries (Table 2). Conversely, 63% of low-income countries and 60% of lower-middle-income countries encouraged the provision of brief advice in existing services compared to 56% of high-income countries.

Access to help was rated as being easily available in general practice, pharmacies, hospitals and the internet in high-income countries, but not in any of the other World Bank income categories (Fig. 2).

The availability of NRT, bupropion and varenicline was lower in lower-income countries (Table 3). No low-income countries at all had NRT or bupropion, nor was bupropion available in any lower-middle-income countries. Cytisine was most widely available in upper-middle-income countries (17%), followed by lower-middle-income countries (13%) and low-income countries (5%), and was least available in high-income countries (3%). The perceived affordability of medications showed a similar pattern; it decreased steadily from high- to low-income countries except for cytisine, which was considered affordable in all countries where it was available (Table 4).

#### Relationship between treatment provision and Parties' WHO region

No consistent pattern was observed in treatment provision by WHO region except for Africa, which was generally lower than other regions.

## Discussion

Only a minority of countries had in place the key infrastructure needed to offer cessation support to tobacco users. A small majority reported promoting cessation through mass media campaigns and encouraging brief advice in existing services. However, cessation help was rated as easily accessible in very few settings indeed, and even then only in high-income countries. A third of countries had no specialized treatment services at all. Availability of medications was limited, and they were frequently perceived to be unaffordable. In general, the provision of cessation support was much lower in lower-income countries.

This study has both strengths and limitations. A key limitation of this study is that it relied upon the knowledge of our contacts and, for the most part, their responses could not be validated, although we made a considerable effort to identify contacts as knowledgeable as possible about tobacco cessation. Where responses were unclear we corresponded with respondents to ensure that the questions had not been misinterpreted and to clarify their responses. With some questions we acknowledge a degree of subjectivity in interpretation of their meaning, but there was a trade-off between attaining as large a sample as possible and keeping the questionnaire to a manageable size. With one question, on medications, we had difficulty interpreting the responses because of the way it was constructed, so we supplemented this with manufacturers' data.

In addition, the proportion of countries running mass media campaigns was higher than we expected; respondents may have included the use of unpaid media and media advocacy as well as paid mass media campaigns when responding to this question. The principal strength of this survey is that it is, to our knowledge, the most extensive, detailed survey of tobacco cessation support conducted to date, with responses from 68% of FCTC Parties.

### Basic cessation infrastructure

The FCTC Article 14 guidelines (the FCTC guidelines) recommend a stepwise approach to promoting tobacco cessation and developing support, and they also recommend prioritizing approaches to providing support that are broad-reach and low-cost 2. Our results show that most countries have not yet put into place the most basic infrastructure for promoting cessation and treatment, including an official responsible for cessation, an official national strategy and national treatment guidelines, this last a treaty obligation.

The FCTC guidelines also stress that addressing tobacco use in health care workers is a priority. Health-care workers are health role models 8,9, tobacco use in this group undermines anti-tobacco public health messages 9, and makes health care workers less likely to encourage their patients to quit 8,9. Rates of tobacco use by health professionals are as high as 40% in some countries 10,11, yet fewer than half of countries offered support to this group.

Routine recording of patients' tobacco use in medical notes is essential if health professionals are to identify and advise all tobacco users. The FCTC guidelines stress that *all* countries should implement this measure; fewer than a quarter have done so.

Few countries had national training standards or a system for monitoring service provision. This may be partly because of cost, and is an area where high-income countries might well be able to support lower income countries, as is encouraged by the FCTC itself.

### National cessation support system

More than half of countries ran mass media campaigns promoting cessation. Such campaigns are crucial to combat the effects of tobacco industry marketing 8 as they influence attitudes to tobacco use, discourage the initiation of tobacco use, motivate quit attempts, generate demand for cessation support and inform people as to what support is available 12.

#### Brief advice

As noted above, the FCTC guidelines recommend integrating brief advice into the health care system as a high priority 2, and state that all countries should be doing this. The 2009 MPOWER report 13 recommends that all countries should provide brief advice, quitlines and access to low-cost medications. Brief advice is one of the most cost-effective disease prevention interventions 14, and its incorporation into health care systems such as primary care would achieve good population coverage at relatively low cost. Repeated exposure to cessation messages in health care settings has also been shown to increase the success of quit attempts significantly 15. Only just over half of countries surveyed promoted brief advice in existing services; we believe this intervention should be prioritized urgently. Delivery of brief advice is likely to be linked to the recording of tobacco use in patients' notes. If tobacco use is recorded and updated regularly in patient notes this will act as a prompt to offer cessation advice on a regular basis.

#### Quitlines

Just over a third of countries had quitlines. Overall, the majority were free and were answered by people most of the time, but only just over half offered multiple sessions, with counsellors calling back to offer ongoing support. Although quitlines are much cheaper than face-to-face specialist treatment, these results illustrate the greater provision of cessation support in higher-income countries, and possibly suggest that for low-income countries even quitlines may be perceived to be relatively expensive 16. Even lower-cost options such as the use of text messaging (to support tobacco users trying to quit) deserve study 17.

#### Specialist treatment facilities

Specialized tobacco treatment services were available widely in very few countries, and a third (63% in low-income countries) had no treatment services at all. The relatively high cost of establishing specialist services is almost certainly a deterrent to many countries, which is why the FCTC guidelines urge countries so strongly not to develop such services until lower-cost broader-reach interventions have been established. A number of middle-income countries 18 have established a system of specialized treatment clinics which would be extremely difficult to expand even if they could afford to do so. For a country with very limited resources to start with, specialist services are unlikely to be a cost-effective use of these limited resources.

#### Access to help

In the vast majority of countries, very few tobacco users could obtain help easily to stop tobacco use in any settings. Help was available most readily in general/family practice, but even in this setting tobacco users could obtain help easily in only 30% of countries. No other setting reached 25%. Clearly, to expand access to cessation help quickly, countries should follow the FCTC guidelines' recommendations to focus on low-cost broad-reach interventions and on using existing infrastructure, such as primary care, to the maximum extent possible. Access to support on the internet was also low. Given its potential cost-effectiveness this approach merits more research.

#### Access to medications

Cessation attempts using medications are more successful than those made without medications 19–24, thus medications have an important role to play in assisting cessation. However, they were not available in all countries and in many, especially lower-income countries, were perceived as being unaffordable. Unfortunately, most countries' health care systems do not cover the cost of tobacco cessation medications and in some countries even NRT, one of the less expensive medications, is far more expensive than cigarettes 25,26. In our survey, respondents in 70% or more of countries rated NRT and bupropion as easily affordable, but varenicline was judged to be affordable in fewer than half the countries in which it is available. The FCTC guidelines urge countries to look at ways of reducing the cost of medications, including by bulk-buying, for example. Cytisine was rated as being ‘easily affordable to most tobacco users’ in all eight countries where it was available. One was high-income, four were upper-middle-income countries, two were lower-middle-income countries and one was low-income. The fact that a course of cytisine is reported to cost US$15 or less 27,28 suggests that more widespread licensing of cytisine could make it affordable to millions of tobacco users currently unable to afford medications.

#### Relationship between treatment provision and Parties' World Bank income level

Perhaps not surprisingly, almost all aspects of cessation provision were more common in higher-income countries, with the high availability of help from the internet probably reflecting better internet access in these countries. A comprehensive specialist treatment service covering the whole country, such as that established in England in 1999, can be expensive. The English services were set up with an initial budget of $83 million 29. We know informally that many countries are concerned by the perceived cost of cessation support, and it is because of this that the FCTC guidelines emphasize so strongly starting with affordable, broad-reach approaches, including brief advice and quitlines. Our finding that the promotion of brief advice was more common in lower- than in higher-income countries might suggest that countries are beginning to take this message on board.

#### Relationship between treatment provision and Parties' WHO region

The only consistent finding in treatment provision by WHO region was that provision in Africa was generally lower than in other regions, probably as a result of the predominance of low- and lower-middle-income countries in the region.

## Conclusions

To our knowledge, this is the most extensive survey of tobacco dependence treatment conducted to date, with responses from more than two-thirds of FCTC Parties. Overall, tobacco cessation support and treatment appear to be a low priority for most Parties, especially lower-income countries. The FCTC Article 14 guidelines recommend that before establishing treatment services, countries first put into place measures proposed in other Articles of the FCTC (especially Articles 6, 8, 11, 12 and 13) that are designed to promote cessation and likely to create demand for cessation support. The Article 14 guidelines do, however, acknowledge the need for flexibility, and recommend that brief advice should be implemented in all countries as a priority. There remains much room for improvement in the extent to which low-cost broad-reach interventions can be implemented in low- and middle-income countries, with an emphasis on the stepwise approach advocated by the Article 14 guidelines.

Other areas requiring attention are establishing national coordinating mechanisms, official national strategies and help for health care workers to quit. Finally, tobacco dependence treatment is highly cost-effective, and therefore should be a higher priority in higher-income countries.

Treatment provision is an integral part of wider tobacco control measures being implemented by Parties, as it indicates governments' recognition that tobacco use is addictive, and willingness to provide support to those who genuinely need it. As stated in the FCTC Article 14 guidelines, providing these services increases acceptability of and social support for other tobacco control policies 2.
